# Epilepsy as a Rare Neurologic Manifestation of Oculodentodigitalis Dysplasia

**Published:** 2012

**Authors:** Mohammad BARZEGAR, Mohammad SAYADNASIRI, Aidin TABRIZI

**Affiliations:** 1 Professor of pediatric Neurology, Pediatric Health Research Center, Tabriz University of Medical Sciences, Tabriz, Iran; 2 Assistant professor of Neurology, Department of Neurology, Qazvin University of Medical Sciences, Qazvin, Iran; 3 Pediarician, Pediatric Health Research Center, Tabriz University of Medical Sciences,Tabriz, Iran

**Keywords:** Oculodentodigitalis dysplasia, Epilepsy, Spasticity

## Abstract

Oculodentodigitalis dysplasia (ODDD) is an extremely rare inherited disorder involving the development of the face, eyes, teeth and limbs. In addition, some patients develop neurological problems mostly a spastic paraparesis associated with white matter abnormalities on magnetic resonance imaging.

This report describes a patient with epilepsy, a rare neurologic manifestation of this syndrome.

## Introduction

Oculodentodigitalis dysplasia (ODDD) Mandolin Inheritance in Man (MIM 16200) is a rare and mainly autosomal dominant hereditary disorder with variable expression (1).ODDD is due to mutations in the GJA1 gene located on human chromosomes 6q22-q24 and encodes the gap junction protein Connexin 43 (2). This is a condition with multiple involved parts of the body; mainly the eyes as microphthalmia, microcornea, glaucoma, cataracts and optic atrophy; teeth as enamel hypoplasia, caries, permanent dentition and delayed tooth eruption; and the fingers as bilateral type III syndactyly of the fourth and fifth fingers and campodactyly and clinodactyly of the fifth fingers. Craniofacial abnormalities include a depressed nasal bridge, thin nose with hypoplastic alae nasi, small anteverted nostrils, prominent columella and microcephaly. Fine, dry, thin, sparse and slow growing hair (hypotrichosis) and brittle nails are also prominent (3-5).

In some reports, neurologic manifestations of this syndrome have been noted. Spastic paraparesis, subcortical white matter lesion and basal ganglia changes on MRI are the most frequent findings, but other abnormalities such as gaze palsy, bladder and bowel dysfunction, visual loss, nystagmus, ataxia, muscle weakness and paresthesia have also been reported in the literature (6-7). 

Epilepsy as a manifestation of ODDD is extremely rare and has only been reported in few cases (8). This report describes a case of ODDD with epilepsy. 

## Case report

**Fig 1:**
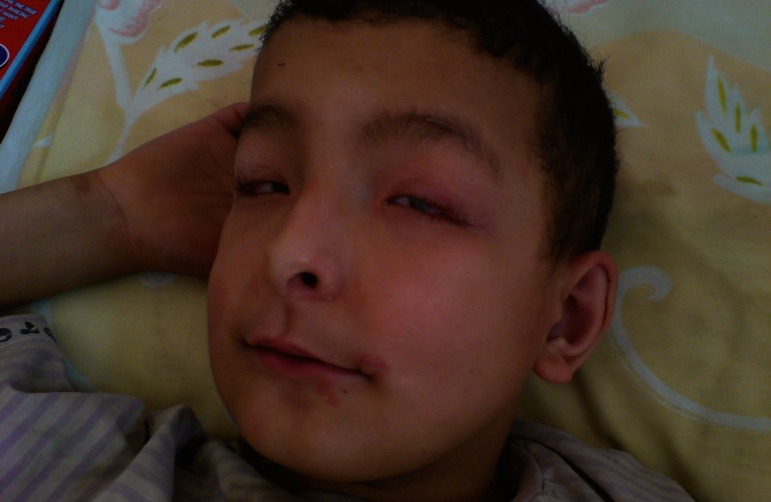
Examination of the face revealed a long and thin nose with hypoplasia of nasal alae and anteverted nostrils

**Fig 2:**
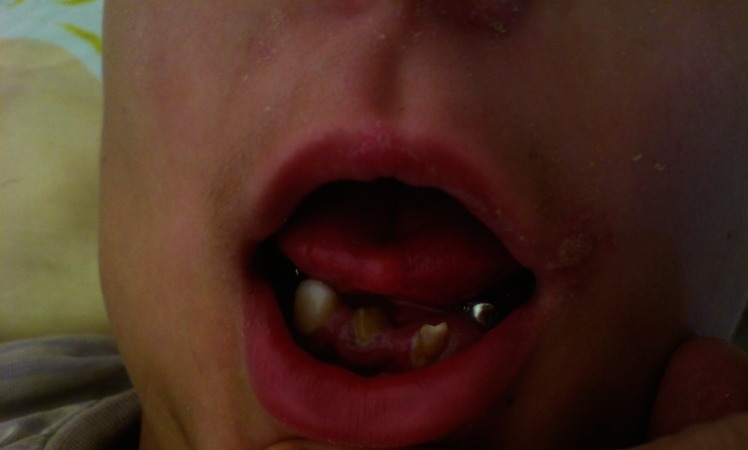
Oral cavity examination revealed yellowish color of the teeth with carries and smaller than normal and atypically-shaped teeth with arrested development appearance

**Fig3:**
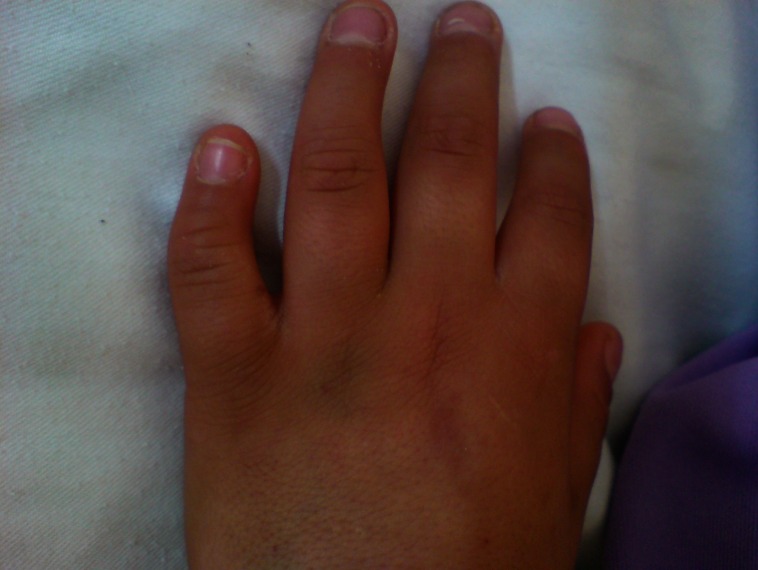
Camptodactyly and hypoplasia of the mid phalanx of the fifth fingers

This 11-year-old boy was the second child of healthy unrelated parents. At the time of birth, his father and mother were 30 and 25 years old, respectively. He was a term newborn and his prenatal as well as perinatal period history were uncomplicated, except for mild hyperbilirubinemia (15 mg/dl of total bilirubin) at day 4 probably due to breast milk which improved with simple phototherapy. His early psychomotor development was normal. He completed the first and second level of elementary school with good scores. He developed one episode of generalized tonic-clonic seizure at age eight. It was repeated several days later; therefore, he was admitted to our hospital and phenytoin was started and the seizures were controlled. At this time precise physical and neurological examination unfolded clinical features of ODDD. Examination of the face revealed a long and thin nose with hypoplasia of nasal alae and anteverted nostrils (Fig 1). Ophthalmic examination revealed microphthalmos with microcornea (Fig 1), decreased visual acuity and glaucoma. Hearing assessment was normal. Oral cavity examination revealed yellowish color of the teeth with carries and smaller than normal and atypically-shaped teeth with arrested development appearance (Fig 2). On skeletal examination, his hand revealed evidence of webbing of the skin (syndactyly) between the fourth and fifth fingers. There was also evidence of camptodactyly and hypoplasia of the mid phalanx of the fifth fingers (Fig 3). Both parents were carefully examined and showed no stigmata suggestive of ODDD. His school performance was gradually deteriorated after the episode of seizure at age eight. One year later he complained of gait problem that has progressively worsened. There was spasticity of both legs with hyper-reflexia and extensor plantar responses. Gait examination showed spastic paraparesis. 

**Fig 4(A, B).**
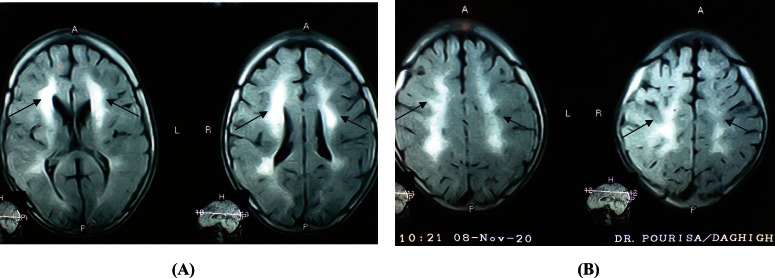
On brain magnetic resonance imaging (MRI), symmetrical confluent hypersignal areas of subcortical white matter were evident with decreased signal of both basal ganglia and pachygyria

On brain magnetic resonance imaging (MRI), symmetrical confluent hypersignal areas of subcortical white matter were evident with decreased signal of both basal ganglia and pachygyria (Fig 4 and 5). 

Unfortunately, genetic examination was not available in our country at that time. The last admission of the patient at the age of 10 years, was due to status epilepticus, which was controlled with antiepileptic drugs. Repeated electroencephalograms (EEG) showed slow background

for this age with epileptiform discharges. Now he takes phenobarbital and carbamazepine for epilepsy, baclofen for spasticity, timolol for glaucoma and physiotherapy for gait problem.

## Discussion

In 1890, Brailey described a 18-year-old girl with bilateral microphthalmus, glaucoma, and abnormal development of the teeth. After 30 years, Lohmann described two unrelated 10-year-old girls with a syndrome of microphthalmia, hypoplastic nasal alae, syndactyly, bilateral campatodactyly of the fifth finger and enamel hypoplasia. In 1957, this syndrome was first described as an individual clinical entity by Meyer-Schwickerath et al.; he introduced the term ‘dysplasia oculo-dentodigitalis’ and in 1963, Gorlin et al. summarized the six known cases and defined the syndrome (9).

ODDD is a rare disease and by our knowledge, 243 cases of ODDD have been reported in the literatures from 1890 to date (10). A wide range of neurological symptoms have been described in ODDD, due to the different interests of authors of most reports, the prevalence of these manifestations have not been determined exactly. In many of the reported patients neurologic symptoms have been scarce and have occasionally been overlooked by authors, but on the other hand, in some cases, CNS involvement was the prominent feature. In a recent study on 73 pediatric patients with ODDD, 29% of the cases had neurologic symptoms (11). More frequent reported clinical and paraclinical neurological findings include dysarthria, neurogenic bladder disturbances, spastic paraparesis, ataxia, anterior tibial muscle weakness, seizures and abnormal cerebral white matter on MRI study (6-8,12-13).

The most prominent MRI abnormality of ODDD is change of the subcortical white matter. White matter abnormality is an explanation for some of the neurological findings of these patients such as spasticity which is correlated with the disease severity (3). Other reported imaging abnormalities on MRI study included hypointensity in the globus pallidus, substantia nigra, red nucleus, thalamus and also the cortex that may be due to iron deposition (14).

Although our patient developed multiple neurological manifestations including spastic paraparesis and subsequently gait disturbance and white matter abnormities on MRI study, we emphasize on epilepsy as a rare finding of ODDD which was the presenting feature of our case. Epilepsy has been described in few reports (8). Amador et al. report a 4-generation Hispanic family with oculodentodigital dysplasia whose members were found to have typical phenotypic characteristics of this disorder, as well as a variable expression of neurologic manifestations in multiple generations ranging from a mild spastic gait to moderate to severe spastic tetraparesis/quadriplegia with epilepsy and an abnormal brain and spinal cord magnetic resonance imaging result (15).

In our patient, MRI showed pachygyria that may be a logical explanation for some rare manifestations such as epilepsy.

Eventually, this report emphasizes that the CNS pathology of ODDD is not limited to the white matter area and ongoing cortical damage is the probable cause of additional neurological manifestations such as epilepsy.

**In conclusion, **oculodentodigital dysplasia is associated with a broad range of neurologic manifestations, so detailed clinical examination and paraclinical evaluations (such as EEG and MRI) of the neurological system are recommended for all ODDD patients.
